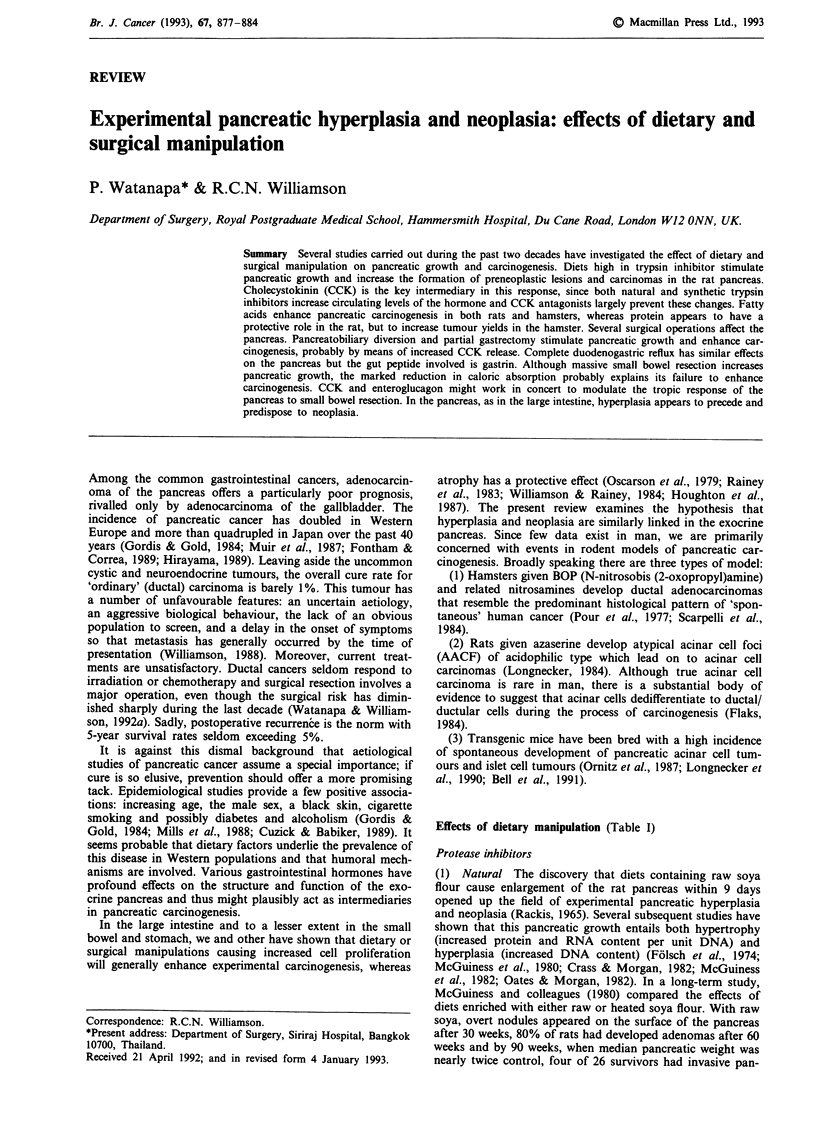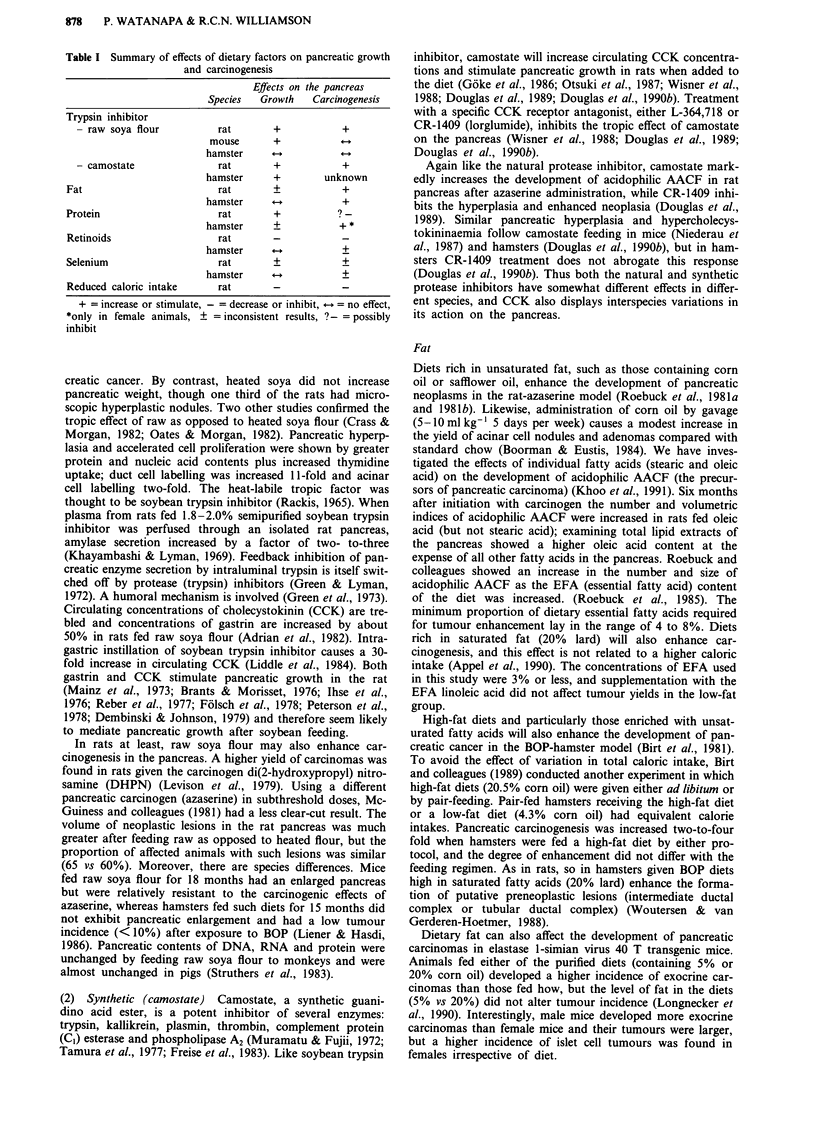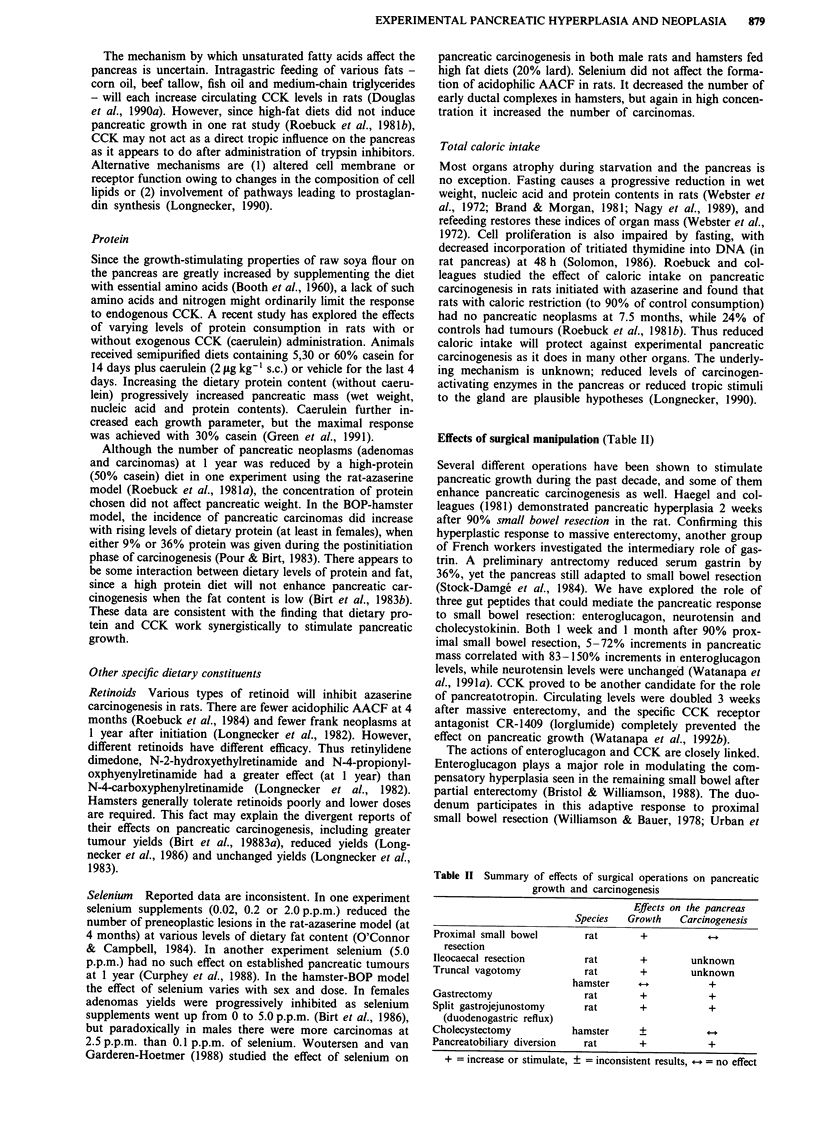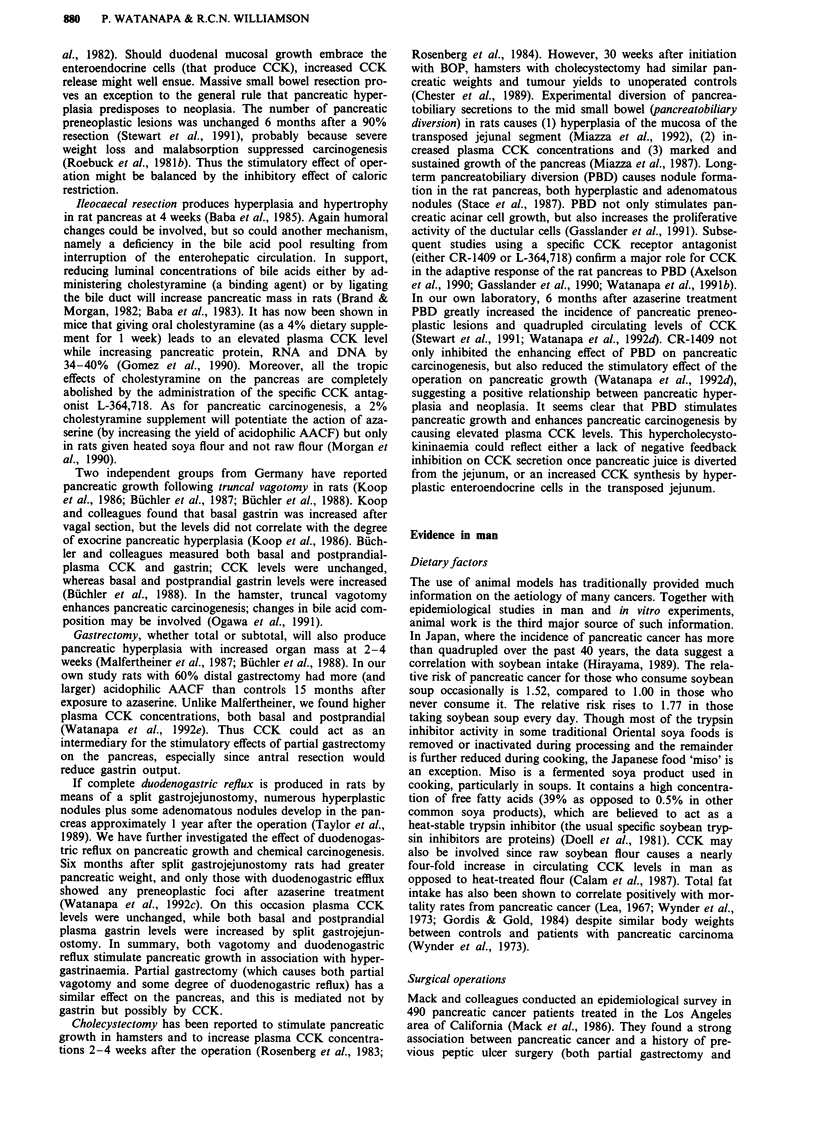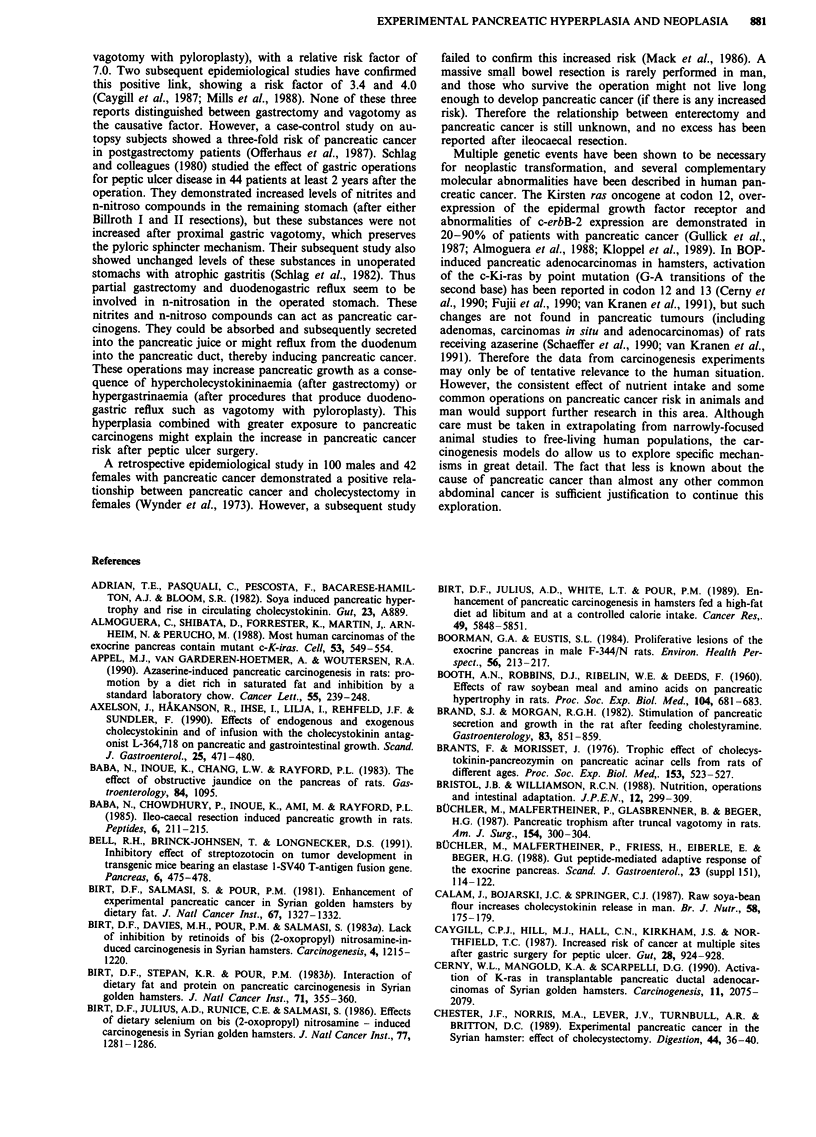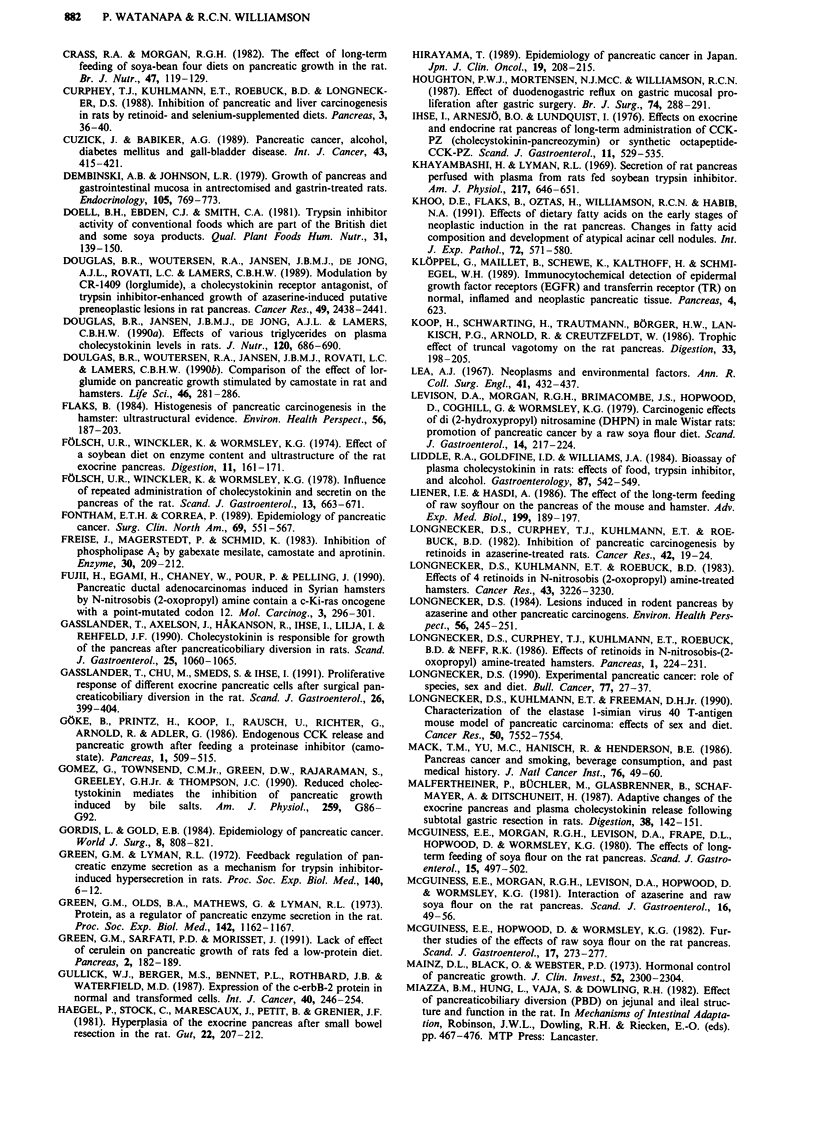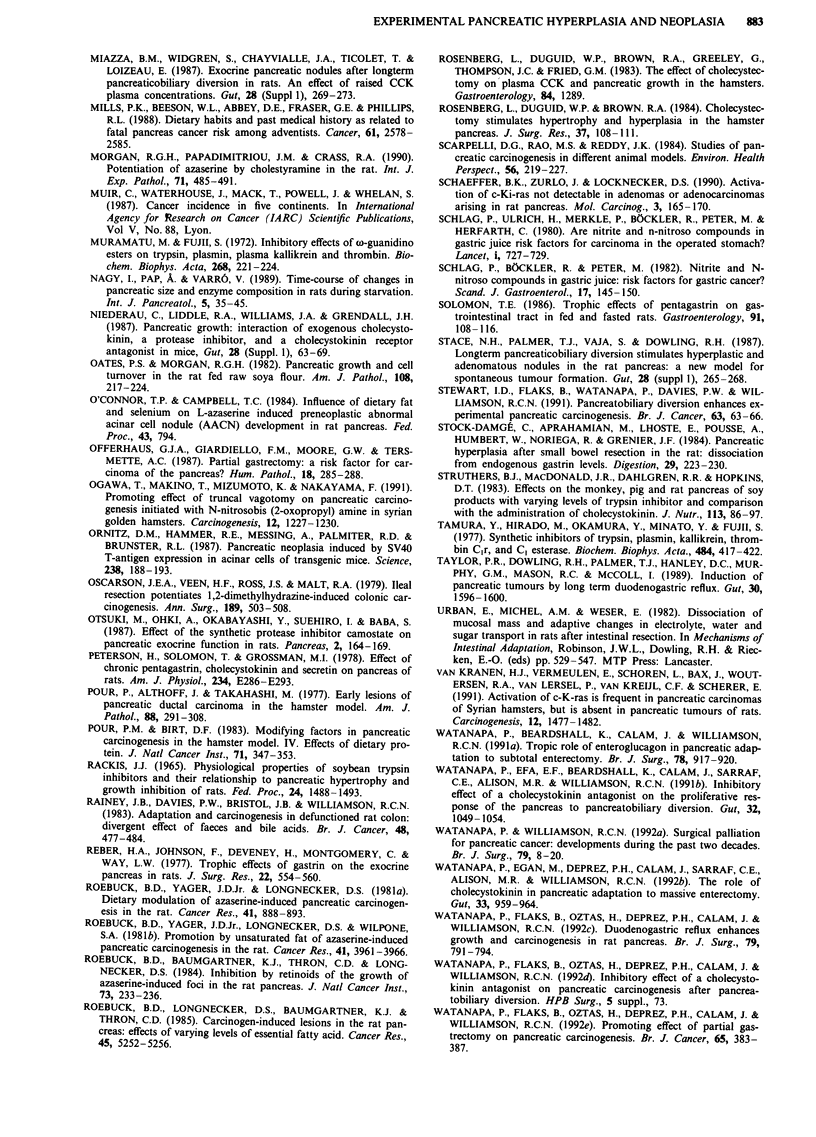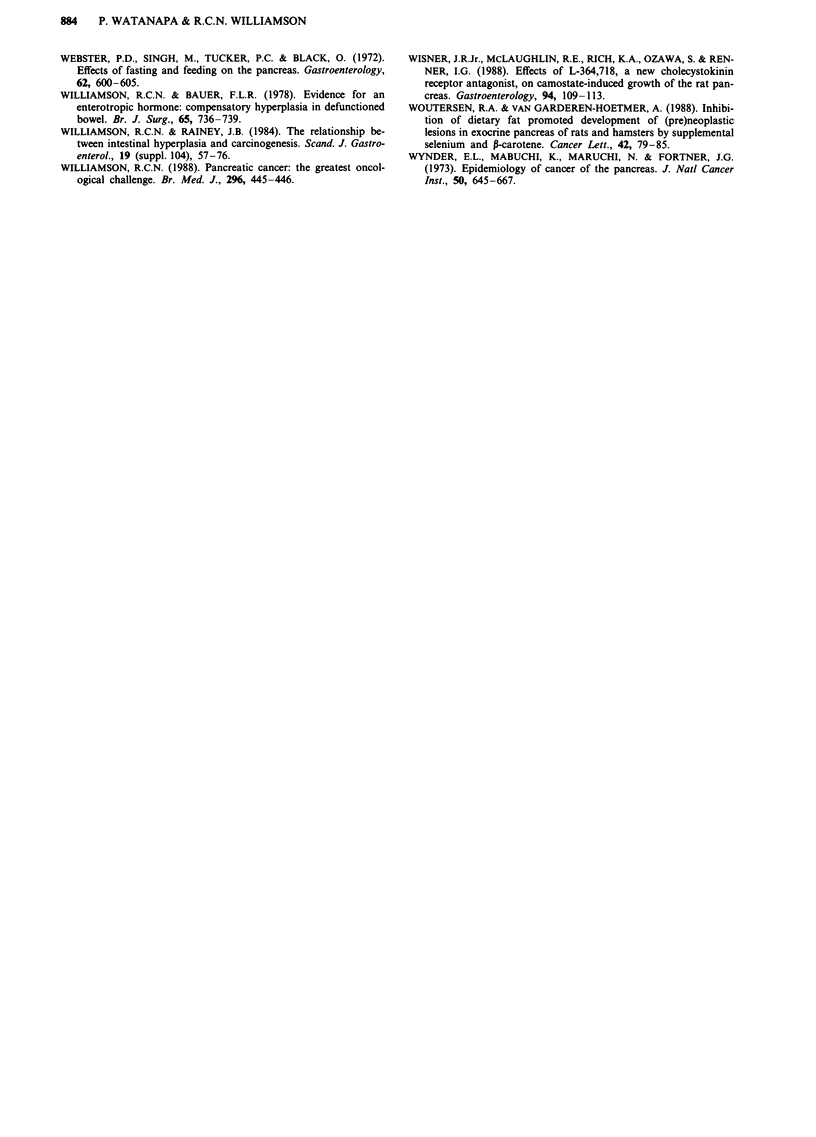# Experimental pancreatic hyperplasia and neoplasia: effects of dietary and surgical manipulation.

**DOI:** 10.1038/bjc.1993.165

**Published:** 1993-05

**Authors:** P. Watanapa, R. C. Williamson

**Affiliations:** Department of Surgery, Royal Postgraduate Medical School, Hammersmith Hospital, London, UK.

## Abstract

Several studies carried out during the past two decades have investigated the effect of dietary and surgical manipulation on pancreatic growth and carcinogenesis. Diets high in trypsin inhibitor stimulate pancreatic growth and increase the formation of preneoplastic lesions and carcinomas in the rat pancreas. Cholecystokinin (CCK) is the key intermediary in this response, since both natural and synthetic trypsin inhibitors increase circulating levels of the hormone and CCK antagonists largely prevent these changes. Fatty acids enhance pancreatic carcinogenesis in both rats and hamsters, whereas protein appears to have a protective role in the rat, but to increase tumour yields in the hamster. Several surgical operations affect the pancreas. Pancreatobiliary diversion and partial gastrectomy stimulate pancreatic growth and enhance carcinogenesis, probably by means of increased CCK release. Complete duodenogastric reflux has similar effects on the pancreas but the gut peptide involved is gastrin. Although massive small bowel resection increases pancreatic growth, the marked reduction in caloric absorption probably explains its failure to enhance carcinogenesis. CCK and enteroglucagon might work in concert to modulate the tropic response of the pancreas to small bowel resection. In the pancreas, as in the large intestine, hyperplasia appears to precede and predispose to neoplasia.


Br. J. Cancer (1993), 67, 877-884 © Macmillan Press Ltd., 1993

REVIEW

### Experimental pancreatic hyperplasia and neoplasia: effects of dietary and surgical manipulation

P. Watanapa* & R.C.N. Williamson

*Department of Surgery, Royal Postgraduate Medical School, Hammersmith Hospital, Du Cane Road, London W12 0NN, UK.*

**Summary** Several studies carried out during the past two decades have investigated the effect of dietary and surgical manipulation on pancreatic growth and carcinogenesis. Diets high in trypsin inhibitor stimulate pancreatic growth and increase the formation of preneoplastic lesions and carcinomas in the rat pancreas. Cholecystokinin (CCK) is the key intermediary in this response, since both natural and synthetic trypsin inhibitors increase circulating levels of the hormone and CCK antagonists largely prevent these changes. Fatty acids enhance pancreatic carcinogenesis in both rats and hamsters, whereas protein appears to have a protective role in the rat, but to increase tumour yields in the hamster. Several surgical operations affect the pancreas. Pancreatobiliary diversion and partial gastrectomy stimulate pancreatic growth and enhance carcinogenesis, probably by means of increased CCK release. Complete duodenogastric reflux has similar effects on the pancreas but the gut peptide involved is gastrin. Although massive small bowel resection increases pancreatic growth, the marked reduction in caloric absorption probably explains its failure to enhance carcinogenesis. CCK and enteroglucagon might work in concert to modulate the tropic response of the pancreas to small bowel resection. In the pancreas, as in the large intestine, hyperplasia appears to precede and predispose to neoplasia.

Among the common gastrointestinal cancers, adenocarcinoma of the pancreas offers a particularly poor prognosis, rivalled only by adenocarcinoma of the gallbladder. The incidence of pancreatic cancer has doubled in Western Europe and more than quadrupled in Japan over the past 40 years (Gordis & Gold, 1984; Muir *et al.*, 1987; Fontham & Correa, 1989; Hirayama, 1989). Leaving aside the uncommon cystic and neuroendocrine tumours, the overall cure rate for 'ordinary' (ductal) carcinoma is barely 1%. This tumour has a number of unfavourable features: an uncertain aetiology, an aggressive biological behaviour, the lack of an obvious population to screen, and a delay in the onset of symptoms so that metastasis has generally occurred by the time of presentation (Williamson, 1988). Moreover, current treatments are unsatisfactory. Ductal cancers seldom respond to irradiation or chemotherapy and surgical resection involves a major operation, even though the surgical risk has diminished sharply during the last decade (Watanapa & Williamson, 1992*a*). Sadly, postoperative recurrence is the norm with 5-year survival rates seldom exceeding 5%.

It is against this dismal background that aetiological studies of pancreatic cancer assume a special importance; if cure is so elusive, prevention should offer a more promising tack. Epidemiological studies provide a few positive associations: increasing age, the male sex, a black skin, cigarette smoking and possibly diabetes and alcoholism (Gordis & Gold, 1984; Mills *et al.*, 1988; Cuzick & Babiker, 1989). It seems probable that dietary factors underlie the prevalence of this disease in Western populations and that humoral mechanisms are involved. Various gastrointestinal hormones have profound effects on the structure and function of the exocrine pancreas and thus might plausibly act as intermediaries in pancreatic carcinogenesis.

In the large intestine and to a lesser extent in the small bowel and stomach, we and other have shown that dietary or surgical manipulations causing increased cell proliferation will generally enhance experimental carcinogenesis, whereas atrophy has a protective effect (Oscarson *et al.*, 1979; Rainey *et al.*, 1983; Williamson & Rainey, 1984; Houghton *et al.*, 1987). The present review examines the hypothesis that hyperplasia and neoplasia are similarly linked in the exocrine pancreas. Since few data exist in man, we are primarily concerned with events in rodent models of pancreatic carcinogenesis. Broadly speaking there are three types of model:

(1) Hamsters given BOP (N-nitrosobis (2-oxopropyl)amine) and related nitrosamines develop ductal adenocarcinomas that resemble the predominant histological pattern of 'spontaneous' human cancer (Pour *et al.*, 1977; Scarpelli *et al.*, 1984).

(2) Rats given azaserine develop atypical acinar cell foci (AACF) of acidophilic type which lead on to acinar cell carcinomas (Longnecker, 1984). Although true acinar cell carcinoma is rare in man, there is a substantial body of evidence to suggest that acinar cells dedifferentiate to ductal/ductular cells during the process of carcinogenesis (Flaks, 1984).

(3) Transgenic mice have been bred with a high incidence of spontaneous development of pancreatic acinar cell tumours and islet cell tumours (Ornitz *et al.*, 1987; Longnecker *et al.*, 1990; Bell *et al.*, 1991).

### Effects of dietary manipulation (Table I)

### *Protease inhibitors*

(1) *Natural* The discovery that diets containing raw soya flour cause enlargement of the rat pancreas within 9 days opened up the field of experimental pancreatic hyperplasia and neoplasia (Rackis, 1965). Several subsequent studies have shown that this pancreatic growth entails both hypertrophy (increased protein and RNA content per unit DNA) and hyperplasia (increased DNA content) (Fölsch *et al.*, 1974; McGuiness *et al.*, 1980; Crass & Morgan, 1982; McGuiness *et al.*, 1982; Oates & Morgan, 1982). In a long-term study, McGuiness and colleagues (1980) compared the effects of diets enriched with either raw or heated soya flour. With raw soya, overt nodules appeared on the surface of the pancreas after 30 weeks, 80% of rats had developed adenomas after 60 weeks and by 90 weeks, when median pancreatic weight was nearly twice control, four of 26 survivors had invasive pan-

Correspondence: R.C.N. Williamson.
*Present address: Department of Surgery, Siriraj Hospital, Bangkok 10700, Thailand.
Received 21 April 1992; and in revised form 4 January 1993.

Table I Summary of effects of dietary factors on pancreatic growth and carcinogenesis

| | Species | Effects on the pancreas Growth | Carcinogenesis |
|---|---|---|---|
| Trypsin inhibitor | | | |
| – raw soya flour | rat | + | + |
| | mouse | + | ↔ |
| | hamster | ↔ | ↔ |
| – camostate | rat | + | + |
| | hamster | + | unknown |
| Fat | rat | ± | + |
| | hamster | ↔ | + |
| Protein | rat | + | ? – |
| | hamster | ± | + * |
| Retinoids | rat | – | – |
| | hamster | ↔ | ± |
| Selenium | rat | ± | ± |
| | hamster | ↔ | ± |
| Reduced caloric intake | rat | – | – |

+ = increase or stimulate, – = decrease or inhibit, ↔ = no effect, *only in female animals, ± = inconsistent results, ?– = possibly inhibit creatic cancer. By contrast, heated soya did not increase pancreatic weight, though one third of the rats had microscopic hyperplastic nodules. Two other studies confirmed the tropic effect of raw as opposed to heated soya flour (Crass & Morgan, 1982; Oates & Morgan, 1982). Pancreatic hyperplasia and accelerated cell proliferation were shown by greater protein and nucleic acid contents plus increased thymidine uptake; duct cell labelling was increased 11-fold and acinar cell labelling two-fold. The heat-labile tropic factor was thought to be soybean trypsin inhibitor (Rackis, 1965). When plasma from rats fed 1.8–2.0% semipurified soybean trypsin inhibitor was perfused through an isolated rat pancreas, amylase secretion increased by a factor of two- to-three (Khayambashi & Lyman, 1969). Feedback inhibition of pancreatic enzyme secretion by intraluminal trypsin is itself switched off by protease (trypsin) inhibitors (Green & Lyman, 1972). A humoral mechanism is involved (Green *et al.*, 1973). Circulating concentrations of cholecystokinin (CCK) are trebled and concentrations of gastrin are increased by about 50% in rats fed raw soya flour (Adrian *et al.*, 1982). Intragastric instillation of soybean trypsin inhibitor causes a 30-fold increase in circulating CCK (Liddle *et al.*, 1984). Both gastrin and CCK stimulate pancreatic growth in the rat (Mainz *et al.*, 1973; Brants & Morisset, 1976; Ihse *et al.*, 1976; Reber *et al.*, 1977; Fölsch *et al.*, 1978; Peterson *et al.*, 1978; Dembinski & Johnson, 1979) and therefore seem likely to mediate pancreatic growth after soybean feeding.

In rats at least, raw soya flour may also enhance carcinogenesis in the pancreas. A higher yield of carcinomas was found in rats given the carcinogen di(2-hydroxypropyl) nitrosamine (DHPN) (Levison *et al.*, 1979). Using a different pancreatic carcinogen (azaserine) in subthreshold doses, McGuiness and colleagues (1981) had a less clear-cut result. The volume of neoplastic lesions in the rat pancreas was much greater after feeding raw as opposed to heated flour, but the proportion of affected animals with such lesions was similar (65 *vs* 60%). Moreover, there are species differences. Mice fed raw soya flour for 18 months had an enlarged pancreas but were relatively resistant to the carcinogenic effects of azaserine, whereas hamsters fed such diets for 15 months did not exhibit pancreatic enlargement and had a low tumour incidence (<10%) after exposure to BOP (Liener & Hasdi, 1986). Pancreatic contents of DNA, RNA and protein were unchanged by feeding raw soya flour to monkeys and were almost unchanged in pigs (Struthers *et al.*, 1983).

(2) *Synthetic (camostate)* Camostate, a synthetic guanidino acid ester, is a potent inhibitor of several enzymes: trypsin, kallikrein, plasmin, thrombin, complement protein ($C_1$) esterase and phospholipase $A_2$ (Muramatu & Fujii, 1972; Tamura *et al.*, 1977; Freise *et al.*, 1983). Like soybean trypsin inhibitor, camostate will increase circulating CCK concentrations and stimulate pancreatic growth in rats when added to the diet (Göke *et al.*, 1986; Otsuki *et al.*, 1987; Wisner *et al.*, 1988; Douglas *et al.*, 1989; Douglas *et al.*, 1990*b*). Treatment with a specific CCK receptor antagonist, either L-364,718 or CR-1409 (lorglumide), inhibits the tropic effect of camostate on the pancreas (Wisner *et al.*, 1988; Douglas *et al.*, 1989; Douglas *et al.*, 1990*b*).

Again like the natural protease inhibitor, camostate markedly increases the development of acidophilic AACF in rat pancreas after azaserine administration, while CR-1409 inhibits the hyperplasia and enhanced neoplasia (Douglas *et al.*, 1989). Similar pancreatic hyperplasia and hypercholecystokininaemia follow camostate feeding in mice (Niederau *et al.*, 1987) and hamsters (Douglas *et al.*, 1990*b*), but in hamsters CR-1409 treatment does not abrogate this response (Douglas *et al.*, 1990*b*). Thus both the natural and synthetic protease inhibitors have somewhat different effects in different species, and CCK also displays interspecies variations in its action on the pancreas.

*Fat*

Diets rich in unsaturated fat, such as those containing corn oil or safflower oil, enhance the development of pancreatic neoplasms in the rat-azaserine model (Roebuck *et al.*, 1981*a* and 1981*b*). Likewise, administration of corn oil by gavage (5–10 ml $kg^{-1}$ 5 days per week) causes a modest increase in the yield of acinar cell nodules and adenomas compared with standard chow (Boorman & Eustis, 1984). We have investigated the effects of individual fatty acids (stearic and oleic acid) on the development of acidophilic AACF (the precursors of pancreatic carcinoma) (Khoo *et al.*, 1991). Six months after initiation with carcinogen the number and volumetric indices of acidophilic AACF were increased in rats fed oleic acid (but not stearic acid); examining total lipid extracts of the pancreas showed a higher oleic acid content at the expense of all other fatty acids in the pancreas. Roebuck and colleagues showed an increase in the number and size of acidophilic AACF as the EFA (essential fatty acid) content of the diet was increased. (Roebuck *et al.*, 1985). The minimum proportion of dietary essential fatty acids required for tumour enhancement lay in the range of 4 to 8%. Diets rich in saturated fat (20% lard) will also enhance carcinogenesis, and this effect is not related to a higher caloric intake (Appel *et al.*, 1990). The concentrations of EFA used in this study were 3% or less, and supplementation with the EFA linoleic acid did not affect tumour yields in the low-fat group.

High-fat diets and particularly those enriched with unsaturated fatty acids will also enhance the development of pancreatic cancer in the BOP-hamster model (Birt *et al.*, 1981). To avoid the effect of variation in total caloric intake, Birt and colleagues (1989) conducted another experiment in which high-fat diets (20.5% corn oil) were given either *ad libitum* or by pair-feeding. Pair-fed hamsters receiving the high-fat diet or a low-fat diet (4.3% corn oil) had equivalent calorie intakes. Pancreatic carcinogenesis was increased two-to-four fold when hamsters were fed a high-fat diet by either protocol, and the degree of enhancement did not differ with the feeding regimen. As in rats, so in hamsters given BOP diets high in saturated fatty acids (20% lard) enhance the formation of putative preneoplastic lesions (intermediate ductal complex or tubular ductal complex) (Woutersen & van Gerderen-Hoetmer, 1988).

Dietary fat can also affect the development of pancreatic carcinomas in elastase 1-simian virus 40 T transgenic mice. Animals fed either of the purified diets (containing 5% or 20% corn oil) developed a higher incidence of exocrine carcinomas than those fed how, but the level of fat in the diets (5% *vs* 20%) did not alter tumour incidence (Longnecker *et al.*, 1990). Interestingly, male mice developed more exocrine carcinomas than female mice and their tumours were larger, but a higher incidence of islet cell tumours was found in females irrespective of diet.

The mechanism by which unsaturated fatty acids affect the pancreas is uncertain. Intragastric feeding of various fats – corn oil, beef tallow, fish oil and medium-chain triglycerides – will each increase circulating CCK levels in rats (Douglas *et al.*, 1990*a*). However, since high-fat diets did not induce pancreatic growth in one rat study (Roebuck *et al.*, 1981*b*), CCK may not act as a direct tropic influence on the pancreas as it appears to do after administration of trypsin inhibitors. Alternative mechanisms are (1) altered cell membrane or receptor function owing to changes in the composition of cell lipids or (2) involvement of pathways leading to prostaglandin synthesis (Longnecker, 1990).

*Protein*

Since the growth-stimulating properties of raw soya flour on the pancreas are greatly increased by supplementing the diet with essential amino acids (Booth *et al.*, 1960), a lack of such amino acids and nitrogen might ordinarily limit the response to endogenous CCK. A recent study has explored the effects of varying levels of protein consumption in rats with or without exogenous CCK (caerulein) administration. Animals received semipurified diets containing 5,30 or 60% casein for 14 days plus caerulein ($2\ \mu g\ kg^{-1}$ s.c.) or vehicle for the last 4 days. Increasing the dietary protein content (without caerulein) progressively increased pancreatic mass (wet weight, nucleic acid and protein contents). Caerulein further increased each growth parameter, but the maximal response was achieved with 30% casein (Green *et al.*, 1991).

Although the number of pancreatic neoplasms (adenomas and carcinomas) at 1 year was reduced by a high-protein (50% casein) diet in one experiment using the rat-azaserine model (Roebuck *et al.*, 1981*a*), the concentration of protein chosen did not affect pancreatic weight. In the BOP-hamster model, the incidence of pancreatic carcinomas did increase with rising levels of dietary protein (at least in females), when either 9% or 36% protein was given during the postinitiation phase of carcinogenesis (Pour & Birt, 1983). There appears to be some interaction between dietary levels of protein and fat, since a high protein diet will not enhance pancreatic carcinogenesis when the fat content is low (Birt *et al.*, 1983*b*). These data are consistent with the finding that dietary protein and CCK work synergistically to stimulate pancreatic growth.

*Other specific dietary constituents*

*Retinoids* Various types of retinoid will inhibit azaserine carcinogenesis in rats. There are fewer acidophilic AACF at 4 months (Roebuck *et al.*, 1984) and fewer frank neoplasms at 1 year after initiation (Longnecker *et al.*, 1982). However, different retinoids have different efficacy. Thus retinylidene dimedone, N-2-hydroxyethylretinamide and N-4-propionyloxphyenylretinamide had a greater effect (at 1 year) than N-4-carboxyphenylretinamide (Longnecker *et al.*, 1982). Hamsters generally tolerate retinoids poorly and lower doses are required. This fact may explain the divergent reports of their effects on pancreatic carcinogenesis, including greater tumour yields (Birt *et al.*, 19883*a*), reduced yields (Longnecker *et al.*, 1986) and unchanged yields (Longnecker *et al.*, 1983).

*Selenium* Reported data are inconsistent. In one experiment selenium supplements (0.02, 0.2 or 2.0 p.p.m.) reduced the number of preneoplastic lesions in the rat-azaserine model (at 4 months) at various levels of dietary fat content (O'Connor & Campbell, 1984). In another experiment selenium (5.0 p.p.m.) had no such effect on established pancreatic tumours at 1 year (Curphey *et al.*, 1988). In the hamster-BOP model the effect of selenium varies with sex and dose. In females adenomas yields were progressively inhibited as selenium supplements went up from 0 to 5.0 p.p.m. (Birt *et al.*, 1986), but paradoxically in males there were more carcinomas at 2.5 p.p.m. than 0.1 p.p.m. of selenium. Woutersen and van Garderen-Hoetmer (1988) studied the effect of selenium on pancreatic carcinogenesis in both male rats and hamsters fed high fat diets (20% lard). Selenium did not affect the formation of acidophilic AACF in rats. It decreased the number of early ductal complexes in hamsters, but again in high concentration it increased the number of carcinomas.

*Total caloric intake*

Most organs atrophy during starvation and the pancreas is no exception. Fasting causes a progressive reduction in wet weight, nucleic acid and protein contents in rats (Webster *et al.*, 1972; Brand & Morgan, 1981; Nagy *et al.*, 1989), and refeeding restores these indices of organ mass (Webster *et al.*, 1972). Cell proliferation is also impaired by fasting, with decreased incorporation of tritiated thymidine into DNA (in rat pancreas) at 48 h (Solomon, 1986). Roebuck and colleagues studied the effect of caloric intake on pancreatic carcinogenesis in rats initiated with azaserine and found that rats with caloric restriction (to 90% of control consumption) had no pancreatic neoplasms at 7.5 months, while 24% of controls had tumours (Roebuck *et al.*, 1981*b*). Thus reduced caloric intake will protect against experimental pancreatic carcinogenesis as it does in many other organs. The underlying mechanism is unknown; reduced levels of carcinogen-activating enzymes in the pancreas or reduced tropic stimuli to the gland are plausible hypotheses (Longnecker, 1990).

**Effects of surgical manipulation** (Table II)

Several different operations have been shown to stimulate pancreatic growth during the past decade, and some of them enhance pancreatic carcinogenesis as well. Haegel and colleagues (1981) demonstrated pancreatic hyperplasia 2 weeks after 90% *small bowel resection* in the rat. Confirming this hyperplastic response to massive enterectomy, another group of French workers investigated the intermediary role of gastrin. A preliminary antrectomy reduced serum gastrin by 36%, yet the pancreas still adapted to small bowel resection (Stock-Damgé *et al.*, 1984). We have explored the role of three gut peptides that could mediate the pancreatic response to small bowel resection: enteroglucagon, neurotensin and cholecystokinin. Both 1 week and 1 month after 90% proximal small bowel resection, 5–72% increments in pancreatic mass correlated with 83–150% increments in enteroglucagon levels, while neurotensin levels were unchanged (Watanapa *et al.*, 1991*a*). CCK proved to be another candidate for the role of pancreatotropin. Circulating levels were doubled 3 weeks after massive enterectomy, and the specific CCK receptor antagonist CR-1409 (lorglumide) completely prevented the effect on pancreatic growth (Watanapa *et al.*, 1992*b*).

The actions of enteroglucagon and CCK are closely linked. Enteroglucagon plays a major role in modulating the compensatory hyperplasia seen in the remaining small bowel after partial enterectomy (Bristol & Williamson, 1988). The duodenum participates in this adaptive response to proximal small bowel resection (Williamson & Bauer, 1978; Urban *et*

**Table II** Summary of effects of surgical operations on pancreatic growth and carcinogenesis

| | | Effects on the pancreas | |
|---|---|---|---|
| | *Species* | *Growth* | *Carcinogenesis* |
| Proximal small bowel resection | rat | + | ↔ |
| Ileocaecal resection | rat | + | unknown |
| Truncal vagotomy | rat | + | unknown |
| | hamster | ↔ | + |
| Gastrectomy | rat | + | + |
| Split gastrojejunostomy (duodenogastric reflux) | rat | + | + |
| Cholecystectomy | hamster | ± | ↔ |
| Pancreatobiliary diversion | rat | + | + |

+ = increase or stimulate, ± = inconsistent results, ↔ = no effect

*al.*, 1982). Should duodenal mucosal growth embrace the enteroendocrine cells (that produce CCK), increased CCK release might well ensue. Massive small bowel resection proves an exception to the general rule that pancreatic hyperplasia predisposes to neoplasia. The number of pancreatic preneoplastic lesions was unchanged 6 months after a 90% resection (Stewart *et al.*, 1991), probably because severe weight loss and malabsorption suppressed carcinogenesis (Roebuck *et al.*, 1981*b*). Thus the stimulatory effect of operation might be balanced by the inhibitory effect of caloric restriction.

*Ileocaecal resection* produces hyperplasia and hypertrophy in rat pancreas at 4 weeks (Baba *et al.*, 1985). Again humoral changes could be involved, but so could another mechanism, namely a deficiency in the bile acid pool resulting from interruption of the enterohepatic circulation. In support, reducing luminal concentrations of bile acids either by administering cholestyramine (a binding agent) or by ligating the bile duct will increase pancreatic mass in rats (Brand & Morgan, 1982; Baba *et al.*, 1983). It has now been shown in mice that giving oral cholestyramine (as a 4% dietary supplement for 1 week) leads to an elevated plasma CCK level while increasing pancreatic protein, RNA and DNA by 34–40% (Gomez *et al.*, 1990). Moreover, all the tropic effects of cholestyramine on the pancreas are completely abolished by the administration of the specific CCK antagonist L-364,718. As for pancreatic carcinogenesis, a 2% cholestyramine supplement will potentiate the action of azaserine (by increasing the yield of acidophilic AACF) but only in rats given heated soya flour and not raw flour (Morgan *et al.*, 1990).

Two independent groups from Germany have reported pancreatic growth following *truncal vagotomy* in rats (Koop *et al.*, 1986; Büchler *et al.*, 1987; Büchler *et al.*, 1988). Koop and colleagues found that basal gastrin was increased after vagal section, but the levels did not correlate with the degree of exocrine pancreatic hyperplasia (Koop *et al.*, 1986). Büchler and colleagues measured both basal and postprandial-plasma CCK and gastrin; CCK levels were unchanged, whereas basal and postprandial gastrin levels were increased (Büchler *et al.*, 1988). In the hamster, truncal vagotomy enhances pancreatic carcinogenesis; changes in bile acid composition may be involved (Ogawa *et al.*, 1991).

*Gastrectomy*, whether total or subtotal, will also produce pancreatic hyperplasia with increased organ mass at 2–4 weeks (Malfertheiner *et al.*, 1987; Büchler *et al.*, 1988). In our own study rats with 60% distal gastrectomy had more (and larger) acidophilic AACF than controls 15 months after exposure to azaserine. Unlike Malfertheiner, we found higher plasma CCK concentrations, both basal and postprandial (Watanapa *et al.*, 1992*e*). Thus CCK could act as an intermediary for the stimulatory effects of partial gastrectomy on the pancreas, especially since antral resection would reduce gastrin output.

If complete *duodenogastric reflux* is produced in rats by means of a split gastrojejunostomy, numerous hyperplastic nodules plus some adenomatous nodules develop in the pancreas approximately 1 year after the operation (Taylor *et al.*, 1989). We have further investigated the effect of duodenogastric reflux on pancreatic growth and chemical carcinogenesis. Six months after split gastrojejunostomy rats had greater pancreatic weight, and only those with duodenogastric efflux showed any preneoplastic foci after azaserine treatment (Watanapa *et al.*, 1992*c*). On this occasion plasma CCK levels were unchanged, while both basal and postprandial plasma gastrin levels were increased by split gastrojejunostomy. In summary, both vagotomy and duodenogastric reflux stimulate pancreatic growth in association with hypergastrinaemia. Partial gastrectomy (which causes both partial vagotomy and some degree of duodenogastric reflux) has a similar effect on the pancreas, and this is mediated not by gastrin but possibly by CCK.

*Cholecystectomy* has been reported to stimulate pancreatic growth in hamsters and to increase plasma CCK concentrations 2–4 weeks after the operation (Rosenberg *et al.*, 1983; Rosenberg *et al.*, 1984). However, 30 weeks after initiation with BOP, hamsters with cholecystectomy had similar pancreatic weights and tumour yields to unoperated controls (Chester *et al.*, 1989). Experimental diversion of pancreatobiliary secretions to the mid small bowel (*pancreatobiliary diversion*) in rats causes (1) hyperplasia of the mucosa of the transposed jejunal segment (Miazza *et al.*, 1992), (2) increased plasma CCK concentrations and (3) marked and sustained growth of the pancreas (Miazza *et al.*, 1987). Long-term pancreatobiliary diversion (PBD) causes nodule formation in the rat pancreas, both hyperplastic and adenomatous nodules (Stace *et al.*, 1987). PBD not only stimulates pancreatic acinar cell growth, but also increases the proliferative activity of the ductular cells (Gasslander *et al.*, 1991). Subsequent studies using a specific CCK receptor antagonist (either CR-1409 or L-364,718) confirm a major role for CCK in the adaptive response of the rat pancreas to PBD (Axelson *et al.*, 1990; Gasslander *et al.*, 1990; Watanapa *et al.*, 1991*b*). In our own laboratory, 6 months after azaserine treatment PBD greatly increased the incidence of pancreatic preneoplastic lesions and quadrupled circulating levels of CCK (Stewart *et al.*, 1991; Watanapa *et al.*, 1992*d*). CR-1409 not only inhibited the enhancing effect of PBD on pancreatic carcinogenesis, but also reduced the stimulatory effect of the operation on pancreatic growth (Watanapa *et al.*, 1992*d*), suggesting a positive relationship between pancreatic hyperplasia and neoplasia. It seems clear that PBD stimulates pancreatic growth and enhances pancreatic carcinogenesis by causing elevated plasma CCK levels. This hypercholecystokininaemia could reflect either a lack of negative feedback inhibition on CCK secretion once pancreatic juice is diverted from the jejunum, or an increased CCK synthesis by hyperplastic enteroendocrine cells in the transposed jejunum.

### Evidence in man

### *Dietary factors*

The use of animal models has traditionally provided much information on the aetiology of many cancers. Together with epidemiological studies in man and *in vitro* experiments, animal work is the third major source of such information. In Japan, where the incidence of pancreatic cancer has more than quadrupled over the past 40 years, the data suggest a correlation with soybean intake (Hirayama, 1989). The relative risk of pancreatic cancer for those who consume soybean soup occasionally is 1.52, compared to 1.00 in those who never consume it. The relative risk rises to 1.77 in those taking soybean soup every day. Though most of the trypsin inhibitor activity in some traditional Oriental soya foods is removed or inactivated during processing and the remainder is further reduced during cooking, the Japanese food 'miso' is an exception. Miso is a fermented soya product used in cooking, particularly in soups. It contains a high concentration of free fatty acids (39% as opposed to 0.5% in other common soya products), which are believed to act as a heat-stable trypsin inhibitor (the usual specific soybean trypsin inhibitors are proteins) (Doell *et al.*, 1981). CCK may also be involved since raw soybean flour causes a nearly four-fold increase in circulating CCK levels in man as opposed to heat-treated flour (Calam *et al.*, 1987). Total fat intake has also been shown to correlate positively with mortality rates from pancreatic cancer (Lea, 1967; Wynder *et al.*, 1973; Gordis & Gold, 1984) despite similar body weights between controls and patients with pancreatic carcinoma (Wynder *et al.*, 1973).

### *Surgical operations*

Mack and colleagues conducted an epidemiological survey in 490 pancreatic cancer patients treated in the Los Angeles area of California (Mack *et al.*, 1986). They found a strong association between pancreatic cancer and a history of previous peptic ulcer surgery (both partial gastrectomy and vagotomy with pyloroplasty), with a relative risk factor of 7.0. Two subsequent epidemiological studies have confirmed this positive link, showing a risk factor of 3.4 and 4.0 (Caygill *et al.*, 1987; Mills *et al.*, 1988). None of these three reports distinguished between gastrectomy and vagotomy as the causative factor. However, a case-control study on autopsy subjects showed a three-fold risk of pancreatic cancer in postgastrectomy patients (Offerhaus *et al.*, 1987). Schlag and colleagues (1980) studied the effect of gastric operations for peptic ulcer disease in 44 patients at least 2 years after the operation. They demonstrated increased levels of nitrites and n-nitroso compounds in the remaining stomach (after either Billroth I and II resections), but these substances were not increased after proximal gastric vagotomy, which preserves the pyloric sphincter mechanism. Their subsequent study also showed unchanged levels of these substances in unoperated stomachs with atrophic gastritis (Schlag *et al.*, 1982). Thus partial gastrectomy and duodenogastric reflux seem to be involved in n-nitrosation in the operated stomach. These nitrites and n-nitroso compounds can act as pancreatic carcinogens. They could be absorbed and subsequently secreted into the pancreatic juice or might reflux from the duodenum into the pancreatic duct, thereby inducing pancreatic cancer. These operations may increase pancreatic growth as a consequence of hypercholecystokininaemia (after gastrectomy) or hypergastrinaemia (after procedures that produce duodenogastric reflux such as vagotomy with pyloroplasty). This hyperplasia combined with greater exposure to pancreatic carcinogens might explain the increase in pancreatic cancer risk after peptic ulcer surgery.

A retrospective epidemiological study in 100 males and 42 females with pancreatic cancer demonstrated a positive relationship between pancreatic cancer and cholecystectomy in females (Wynder *et al.*, 1973). However, a subsequent study failed to confirm this increased risk (Mack *et al.*, 1986). A massive small bowel resection is rarely performed in man, and those who survive the operation might not live long enough to develop pancreatic cancer (if there is any increased risk). Therefore the relationship between enterectomy and pancreatic cancer is still unknown, and no excess has been reported after ileocaecal resection.

Multiple genetic events have been shown to be necessary for neoplastic transformation, and several complementary molecular abnormalities have been described in human pancreatic cancer. The Kirsten *ras* oncogene at codon 12, overexpression of the epidermal growth factor receptor and abnormalities of c-*erb*B-2 expression are demonstrated in 20–90% of patients with pancreatic cancer (Gullick *et al.*, 1987; Almoguera *et al.*, 1988; Kloppel *et al.*, 1989). In BOP-induced pancreatic adenocarcinomas in hamsters, activation of the c-Ki-ras by point mutation (G-A transitions of the second base) has been reported in codon 12 and 13 (Cerny *et al.*, 1990; Fujii *et al.*, 1990; van Kranen *et al.*, 1991), but such changes are not found in pancreatic tumours (including adenomas, carcinomas *in situ* and adenocarcinomas) of rats receiving azaserine (Schaeffer *et al.*, 1990; van Kranen *et al.*, 1991). Therefore the data from carcinogenesis experiments may only be of tentative relevance to the human situation. However, the consistent effect of nutrient intake and some common operations on pancreatic cancer risk in animals and man would support further research in this area. Although care must be taken in extrapolating from narrowly-focused animal studies to free-living human populations, the carcinogenesis models do allow us to explore specific mechanisms in great detail. The fact that less is known about the cause of pancreatic cancer than almost any other common abdominal cancer is sufficient justification to continue this exploration.

### References

ADRIAN, T.E., PASQUALI, C., PESCOSTA, F., BACARESE-HAMILTON, A.J. & BLOOM, S.R. (1982). Soya induced pancreatic hypertrophy and rise in circulating cholecystokinin. *Gut*, **23**, A889.

ALMOGUERA, C., SHIBATA, D., FORRESTER, K., MARTIN, J,. ARNHEIM, N. & PERUCHO, M. (1988). Most human carcinomas of the exocrine pancreas contain mutant c-*K-iras*. *Cell*, **53**, 549–554.

APPEL, M.J., VAN GARDEREN-HOETMER, A. & WOUTERSEN, R.A. (1990). Azaserine-induced pancreatic carcinogenesis in rats: promotion by a diet rich in saturated fat and inhibition by a standard laboratory chow. *Cancer Lett.*, **55**, 239–248.

AXELSON, J., HÅKANSON, R., IHSE, I., LILJA, I., REHFELD, J.F. & SUNDLER, F. (1990). Effects of endogenous and exogenous cholecystokinin and of infusion with the cholecystokinin antagonist L-364,718 on pancreatic and gastrointestinal growth. *Scand. J. Gastroenterol.*, **25**, 471–480.

BABA, N., INOUE, K., CHANG, L.W. & RAYFORD, P.L. (1983). The effect of obstructive jaundice on the pancreas of rats. *Gastroenterology*, **84**, 1095.

BABA, N., CHOWDHURY, P., INOUE, K., AMI, M. & RAYFORD, P.L. (1985). Ileo-caecal resection induced pancreatic growth in rats. *Peptides*, **6**, 211–215.

BELL, R.H., BRINCK-JOHNSEN, T. & LONGNECKER, D.S. (1991). Inhibitory effect of streptozotocin on tumor development in transgenic mice bearing an elastase 1-SV40 T-antigen fusion gene. *Pancreas*, **6**, 475–478.

BIRT, D.F., SALMASI, S. & POUR, P.M. (1981). Enhancement of experimental pancreatic cancer in Syrian golden hamsters by dietary fat. *J. Natl Cancer Inst.*, **67**, 1327–1332.

BIRT, D.F., DAVIES, M.H., POUR, P.M. & SALMASI, S. (1983*a*). Lack of inhibition by retinoids of bis (2-oxopropyl) nitrosamine-induced carcinogenesis in Syrian hamsters. *Carcinogenesis*, **4**, 1215–1220.

BIRT, D.F., STEPAN, K.R. & POUR, P.M. (1983*b*). Interaction of dietary fat and protein on pancreatic carcinogenesis in Syrian golden hamsters. *J. Natl Cancer Inst.*, **71**, 355–360.

BIRT, D.F., JULIUS, A.D., RUNICE, C.E. & SALMASI, S. (1986). Effects of dietary selenium on bis (2-oxopropyl) nitrosamine – induced carcinogenesis in Syrian golden hamsters. *J. Natl Cancer Inst.*, **77**, 1281–1286.

BIRT, D.F., JULIUS, A.D., WHITE, L.T. & POUR, P.M. (1989). Enhancement of pancreatic carcinogenesis in hamsters fed a high-fat diet ad libitum and at a controlled calorie intake. *Cancer Res,.* **49**, 5848–5851.

BOORMAN, G.A. & EUSTIS, S.L. (1984). Proliferative lesions of the exocrine pancreas in male F-344/N rats. *Environ. Health Perspect.*, **56**, 213–217.

BOOTH, A.N., ROBBINS, D.J., RIBELIN, W.E. & DEEDS, F. (1960). Effects of raw soybean meal and amino acids on pancreatic hypertrophy in rats. *Proc. Soc. Exp. Biol. Med.*, **104**, 681–683.

BRAND, S.J. & MORGAN, R.G.H. (1982). Stimulation of pancreatic secretion and growth in the rat after feeding cholestyramine. *Gastroenterology*, **83**, 851–859.

BRANTS, F. & MORISSET, J. (1976). Trophic effect of cholecystokinin-pancreozymin on pancreatic acinar cells from rats of different ages. *Proc. Soc. Exp. Biol. Med,.* **153**, 523–527.

BRISTOL, J.B. & WILLIAMSON, R.C.N. (1988). Nutrition, operations and intestinal adaptation. *J.P.E.N.*, **12**, 299–309.

BÜCHLER, M., MALFERTHEINER, P., GLASBRENNER, B. & BEGER, H.G. (1987). Pancreatic trophism after truncal vagotomy in rats. *Am. J. Surg.*, **154**, 300–304.

BÜCHLER, M., MALFERTHEINER, P., FRIESS, H., EIBERLE, E. & BEGER, H.G. (1988). Gut peptide-mediated adaptive response of the exocrine pancreas. *Scand. J. Gastroenterol.*, **23** (suppl 151), 114–122.

CALAM, J., BOJARSKI, J.C. & SPRINGER, C.J. (1987). Raw soya-bean flour increases cholecystokinin release in man. *Br. J. Nutr.*, **58**, 175–179.

CAYGILL, C.P.J., HILL, M.J., HALL, C.N., KIRKHAM, J.S. & NORTHFIELD, T.C. (1987). Increased risk of cancer at multiple sites after gastric surgery for peptic ulcer. *Gut*, **28**, 924–928.

CERNY, W.L., MANGOLD, K.A. & SCARPELLI, D.G. (1990). Activation of K-ras in transplantable pancreatic ductal adenocarcinomas of Syrian golden hamsters. *Carcinogenesis*, **11**, 2075–2079.

CHESTER, J.F., NORRIS, M.A., LEVER, J.V., TURNBULL, A.R. & BRITTON, D.C. (1989). Experimental pancreatic cancer in the Syrian hamster: effect of cholecystectomy. *Digestion*, **44**, 36–40.

CRASS, R.A. & MORGAN, R.G.H. (1982). The effect of long-term feeding of soya-bean four diets on pancreatic growth in the rat. *Br. J. Nutr.*, **47**, 119–129.

CURPHEY, T.J., KUHLMANN, E.T., ROEBUCK, B.D. & LONGNECKER, D.S. (1988). Inhibition of pancreatic and liver carcinogenesis in rats by retinoid- and selenium-supplemented diets. *Pancreas*, **3**, 36–40.

CUZICK, J. & BABIKER, A.G. (1989). Pancreatic cancer, alcohol, diabetes mellitus and gall-bladder disease. *Int. J. Cancer*, **43**, 415–421.

DEMBINSKI, A.B. & JOHNSON, L.R. (1979). Growth of pancreas and gastrointestinal mucosa in antrectomised and gastrin-treated rats. *Endocrinology*, **105**, 769–773.

DOELL, B.H., EBDEN, C.J. & SMITH, C.A. (1981). Trypsin inhibitor activity of conventional foods which are part of the British diet and some soya products. *Qual. Plant Foods Hum. Nutr.*, **31**, 139–150.

DOUGLAS, B.R., WOUTERSEN, R.A., JANSEN, J.B.M.J., DE JONG, A.J.L., ROVATI, L.C. & LAMERS, C.B.H.W. (1989). Modulation by CR-1409 (lorglumide), a cholecystokinin receptor antagonist, of trypsin inhibitor-enhanced growth of azaserine-induced putative preneoplastic lesions in rat pancreas. *Cancer Res.*, **49**, 2438–2441.

DOUGLAS, B.R., JANSEN, J.B.M.J., DE JONG, A.J.L. & LAMERS, C.B.H.W. (1990*a*). Effects of various triglycerides on plasma cholecystokinin levels in rats. *J. Nutr.*, **120**, 686–690.

DOULGAS, B.R., WOUTERSEN, R.A., JANSEN, J.B.M.J., ROVATI, L.C. & LAMERS, C.B.H.W. (1990*b*). Comparison of the effect of lorglumide on pancreatic growth stimulated by camostate in rat and hamsters. *Life Sci.*, **46**, 281–286.

FLAKS, B. (1984). Histogenesis of pancreatic carcinogenesis in the hamster: ultrastructural evidence. *Environ. Health Perspect.*, **56**, 187–203.

FÖLSCH, U.R., WINCKLER, K. & WORMSLEY, K.G. (1974). Effect of a soybean diet on enzyme content and ultrastructure of the rat exocrine pancreas. *Digestion*, **11**, 161–171.

FÖLSCH, U.R., WINCKLER, K. & WORMSLEY, K.G. (1978). Influence of repeated administration of cholecystokinin and secretin on the pancreas of the rat. *Scand. J. Gastroenterol.*, **13**, 663–671.

FONTHAM, E.T.H. & CORREA, P. (1989). Epidemiology of pancreatic cancer. *Surg. Clin. North Am.*, **69**, 551–567.

FREISE, J., MAGERSTEDT, P. & SCHMID, K. (1983). Inhibition of phospholipase $A_2$ by gabexate mesilate, camostate and aprotinin. *Enzyme*, **30**, 209–212.

FUJII, H., EGAMI, H., CHANEY, W., POUR, P. & PELLING, J. (1990). Pancreatic ductal adenocarcinomas induced in Syrian hamsters by N-nitrosobis (2-oxopropyl) amine contain a c-Ki-ras oncogene with a point-mutated codon 12. *Mol. Carcinog.*, **3**, 296–301.

GASSLANDER, T., AXELSON, J., HÅKANSON, R., IHSE, I., LILJA, I. & REHFELD, J.F. (1990). Cholecystokinin is responsible for growth of the pancreas after pancreaticobiliary diversion in rats. *Scand. J. Gastroenterol.*, **25**, 1060–1065.

GASSLANDER, T., CHU, M., SMEDS, S. & IHSE, I. (1991). Proliferative response of different exocrine pancreatic cells after surgical pancreaticobiliary diversion in the rat. *Scand. J. Gastroenterol.*, **26**, 399–404.

GÖKE, B., PRINTZ, H., KOOP, I., RAUSCH, U., RICHTER, G., ARNOLD, R. & ADLER, G. (1986). Endogenous CCK release and pancreatic growth after feeding a proteinase inhibitor (camostate). *Pancreas*, **1**, 509–515.

GOMEZ, G., TOWNSEND, C.M.JR., GREEN, D.W., RAJARAMAN, S., GREELEY, G.H.JR. & THOMPSON, J.C. (1990). Reduced cholectystokinin mediates the inhibition of pancreatic growth induced by bile salts. *Am. J. Physiol.*, **259**, G86–G92.

GORDIS, L. & GOLD, E.B. (1984). Epidemiology of pancreatic cancer. *World J. Surg.*, **8**, 808–821.

GREEN, G.M. & LYMAN, R.L. (1972). Feedback regulation of pancreatic enzyme secretion as a mechanism for trypsin inhibitor-induced hypersecretion in rats. *Proc. Soc. Exp. Biol. Med.*, **140**, 6–12.

GREEN, G.M., OLDS, B.A., MATHEWS, G. & LYMAN, R.L. (1973). Protein, as a regulator of pancreatic enzyme secretion in the rat. *Proc. Soc. Exp. Biol. Med.*, **142**, 1162–1167.

GREEN, G.M., SARFATI, P.D. & MORISSET, J. (1991). Lack of effect of cerulein on pancreatic growth of rats fed a low-protein diet. *Pancreas*, **2**, 182–189.

GULLICK, W.J., BERGER, M.S., BENNET, P.L., ROTHBARD, J.B. & WATERFIELD, M.D. (1987). Expression of the c-erbB-2 protein in normal and transformed cells. *Int. J. Cancer*, **40**, 246–254.

HAEGEL, P., STOCK, C., MARESCAUX, J., PETIT, B. & GRENIER, J.F. (1981). Hyperplasia of the exocrine pancreas after small bowel resection in the rat. *Gut*, **22**, 207–212.

HIRAYAMA, T. (1989). Epidemiology of pancreatic cancer in Japan. *Jpn. J. Clin. Oncol.*, **19**, 208–215.

HOUGHTON, P.W.J., MORTENSEN, N.J.MCC. & WILLIAMSON, R.C.N. (1987). Effect of duodenogastric reflux on gastric mucosal proliferation after gastric surgery. *Br. J. Surg.*, **74**, 288–291.

IHSE, I., ARNESJÖ, B.O. & LUNDQUIST, I. (1976). Effects on exocrine and endocrine rat pancreas of long-term administration of CCK-PZ (cholecystokinin-pancreozymin) or synthetic octapeptide-CCK-PZ. *Scand. J. Gastroenterol.*, **11**, 529–535.

KHAYAMBASHI, H. & LYMAN, R.L. (1969). Secretion of rat pancreas perfused with plasma from rats fed soybean trypsin inhibitor. *Am. J. Physiol.*, **217**, 646–651.

KHOO, D.E., FLAKS, B., OZTAS, H., WILLIAMSON, R.C.N. & HABIB, N.A. (1991). Effects of dietary fatty acids on the early stages of neoplastic induction in the rat pancreas. Changes in fatty acid composition and development of atypical acinar cell nodules. *Int. J. Exp. Pathol.*, **72**, 571–580.

KLÖPPEL, G., MAILLET, B., SCHEWE, K., KALTHOFF, H. & SCHMIEGEL, W.H. (1989). Immunocytochemical detection of epidermal growth factor receptors (EGFR) and transferrin receptor (TR) on normal, inflamed and neoplastic pancreatic tissue. *Pancreas*, **4**, 623.

KOOP, H., SCHWARTING, H., TRAUTMANN., BÖRGER, H.W., LANKISCH, P.G., ARNOLD, R. & CREUTZFELDT, W. (1986). Trophic effect of truncal vagotomy on the rat pancreas. *Digestion*, **33**, 198–205.

LEA, A.J. (1967). Neoplasms and environmental factors. *Ann. R. Coll. Surg. Engl.*, **41**, 432–437.

LEVISON, D.A., MORGAN, R.G.H., BRIMACOMBE, J.S., HOPWOOD, D., COGHILL, G. & WORMSLEY, K.G. (1979). Carcinogenic effects of di (2-hydroxypropyl) nitrosamine (DHPN) in male Wistar rats: promotion of pancreatic cancer by a raw soya flour diet. *Scand. J. Gastroenterol.*, **14**, 217–224.

LIDDLE, R.A., GOLDFINE, I.D. & WILLIAMS, J.A. (1984). Bioassay of plasma cholecystokinin in rats: effects of food, trypsin inhibitor, and alcohol. *Gastroenterology*, **87**, 542–549.

LIENER, I.E. & HASDI, A. (1986). The effect of the long-term feeding of raw soyflour on the pancreas of the mouse and hamster. *Adv. Exp. Med. Biol.*, **199**, 189–197.

LONGNECKER, D.S., CURPHEY, T.J., KUHLMANN, E.T & ROEBUCK, B.D. (1982). Inhibition of pancreatic carcinogenesis by retinoids in azaserine-treated rats. *Cancer Res.*, **42**, 19–24.

LONGNECKER, D.S., KUHLMANN, E.T. & ROEBUCK, B.D. (1983). Effects of 4 retinoids in N-nitrosobis (2-oxopropyl) amine-treated hamsters. *Cancer Res.*, **43**, 3226–3230.

LONGNECKER, D.S. (1984). Lesions induced in rodent pancreas by azaserine and other pancreatic carcinogens. *Environ. Health Perspect.*, **56**, 245–251.

LONGNECKER, D.S., CURPHEY, T.J., KUHLMANN, E.T., ROEBUCK, B.D. & NEFF, R.K. (1986). Effects of retinoids in N-nitrosobis-(2-oxopropyl) amine-treated hamsters. *Pancreas*, **1**, 224–231.

LONGNECKER, D.S. (1990). Experimental pancreatic cancer: role of species, sex and diet. *Bull. Cancer*, **77**, 27–37.

LONGNECKER, D.S., KUHLMANN, E.T. & FREEMAN, D.H.JR. (1990). Characterization of the elastase 1-simian virus 40 T-antigen mouse model of pancreatic carcinoma: effects of sex and diet. *Cancer Res.*, **50**, 7552–7554.

MACK, T.M., YU, M.C., HANISCH, R. & HENDERSON, B.E. (1986). Pancreas cancer and smoking, beverage consumption, and past medical history. *J. Natl Cancer Inst.*, **76**, 49–60.

MALFERTHEINER, P., BÜCHLER, M., GLASBRENNER, B., SCHAFMAYER, A. & DITSCHUNEIT, H. (1987). Adaptive changes of the exocrine pancreas and plasma cholecystokinin release following subtotal gastric resection in rats. *Digestion*, **38**, 142–151.

MCGUINESS, E.E., MORGAN, R.G.H., LEVISON, D.A., FRAPE, D.L., HOPWOOD, D. & WORMSLEY, K.G. (1980). The effects of long-term feeding of soya flour on the rat pancreas. *Scand. J. Gastroenterol.*, **15**, 497–502.

MCGUINESS, E.E., MORGAN, R.G.H., LEVISON, D.A., HOPWOOD, D. & WORMSLEY, K.G. (1981). Interaction of azaserine and raw soya flour on the rat pancreas. *Scand. J. Gastroenterol.*, **16**, 49–56.

MCGUINESS, E.E., HOPWOOD, D. & WORMSLEY, K.G. (1982). Further studies of the effects of raw soya flour on the rat pancreas. *Scand. J. Gastroenterol.*, **17**, 273–277.

MAINZ, D.L., BLACK, O. & WEBSTER, P.D. (1973). Hormonal control of pancreatic growth. *J. Clin. Invest.*, **52**, 2300–2304.

MIAZZA, B.M., HUNG, L., VAJA, S. & DOWLING, R.H. (1982). Effect of pancreaticobiliary diversion (PBD) on jejunal and ileal structure and function in the rat. In *Mechanisms of Intestinal Adaptation*, Robinson, J.W.L., Dowling, R.H. & Riecken, E.-O. (eds). pp. 467–476. MTP Press: Lancaster.

MIAZZA, B.M., WIDGREN, S., CHAYVIALLE, J.A., TICOLET, T. & LOIZEAU, E. (1987). Exocrine pancreatic nodules after longterm pancreaticobiliary diversion in rats. An effect of raised CCK plasma concentrations. *Gut*, **28** (Suppl 1), 269–273.

MILLS, P.K., BEESON, W.L., ABBEY, D.E., FRASER, G.E. & PHILLIPS, R.L. (1988). Dietary habits and past medical history as related to fatal pancreas cancer risk among adventists. *Cancer*, **61**, 2578–2585.

MORGAN, R.G.H., PAPADIMITRIOU, J.M. & CRASS, R.A. (1990). Potentiation of azaserine by cholestyramine in the rat. *Int. J. Exp. Pathol.*, **71**, 485–491.

MUIR, C., WATERHOUSE, J., MACK, T., POWELL, J. & WHELAN, S. (1987). Cancer incidence in five continents. In *International Agency for Research on Cancer (IARC) Scientific Publications*, Vol V, No. 88, Lyon.

MURAMATU, M. & FUJII, S. (1972). Inhibitory effects of ω-guanidino esters on trypsin, plasmin, plasma kallikrein and thrombin. *Biochem. Biophys. Acta*, **268**, 221–224.

NAGY, I., PAP, Á. & VARRÓ, V. (1989). Time-course of changes in pancreatic size and enzyme composition in rats during starvation. *Int. J. Pancreatol.*, **5**, 35–45.

NIEDERAU, C., LIDDLE, R.A., WILLIAMS, J.A. & GRENDALL, J.H. (1987). Pancreatic growth: interaction of exogenous cholecystokinin, a protease inhibitor, and a cholecystokinin receptor antagonist in mice, *Gut*, **28** (Suppl. 1), 63–69.

OATES, P.S. & MORGAN, R.G.H. (1982). Pancreatic growth and cell turnover in the rat fed raw soya flour. *Am. J. Pathol.*, **108**, 217–224.

O'CONNOR, T.P. & CAMPBELL, T.C. (1984). Influence of dietary fat and selenium on L-azaserine induced preneoplastic abnormal acinar cell nodule (AACN) development in rat pancreas. *Fed. Proc.*, **43**, 794.

OFFERHAUS, G.J.A., GIARDIELLO, F.M., MOORE, G.W. & TERSMETTE, A.C. (1987). Partial gastrectomy: a risk factor for carcinoma of the pancreas? *Hum. Pathol.*, **18**, 285–288.

OGAWA, T., MAKINO, T., MIZUMOTO, K. & NAKAYAMA, F. (1991). Promoting effect of truncal vagotomy on pancreatic carcinogenesis initiated with N-nitrosobis (2-oxopropyl) amine in syrian golden hamsters. *Carcinogenesis*, **12**, 1227–1230.

ORNITZ, D.M., HAMMER, R.E., MESSING, A., PALMITER, R.D. & BRUNSTER, R.L. (1987). Pancreatic neoplasia induced by SV40 T-antigen expression in acinar cells of transgenic mice. *Science*, **238**, 188–193.

OSCARSON, J.E.A., VEEN, H.F., ROSS, J.S. & MALT, R.A. (1979). Ileal resection potentiates 1,2-dimethylhydrazine-induced colonic carcinogenesis. *Ann. Surg.*, **189**, 503–508.

OTSUKI, M., OHKI, A., OKABAYASHI, Y., SUEHIRO, I. & BABA, S. (1987). Effect of the synthetic protease inhibitor camostate on pancreatic exocrine function in rats. *Pancreas*, **2**, 164–169.

PETERSON, H., SOLOMON, T. & GROSSMAN, M.I. (1978). Effect of chronic pentagastrin, cholecystokinin and secretin on pancreas of rats. *Am. J. Physiol.*, **234**, E286–E293.

POUR, P., ALTHOFF, J. & TAKAHASHI, M. (1977). Early lesions of pancreatic ductal carcinoma in the hamster model. *Am. J. Pathol.*, **88**, 291–308.

POUR, P.M. & BIRT, D.F. (1983). Modifying factors in pancreatic carcinogenesis in the hamster model. IV. Effects of dietary protein. *J. Natl Cancer Inst.*, **71**, 347–353.

RACKIS, J.J. (1965). Physiological properties of soybean trypsin inhibitors and their relationship to pancreatic hypertrophy and growth inhibition of rats. *Fed. Proc.*, **24**, 1488–1493.

RAINEY, J.B., DAVIES, P.W., BRISTOL, J.B. & WILLIAMSON, R.C.N. (1983). Adaptation and carcinogenesis in defunctioned rat colon: divergent effect of faeces and bile acids. *Br. J. Cancer*, **48**, 477–484.

REBER, H.A., JOHNSON, F., DEVENEY, H., MONTGOMERY, C. & WAY, L.W. (1977). Trophic effects of gastrin on the exocrine pancreas in rats. *J. Surg. Res.*, **22**, 554–560.

ROEBUCK, B.D., YAGER, J.D.Jr. & LONGNECKER, D.S. (1981*a*). Dietary modulation of azaserine-induced pancreatic carcinogenesis in the rat. *Cancer Res.*, **41**, 888–893.

ROEBUCK, B.D., YAGER, J.D.Jr., LONGNECKER, D.S. & WILPONE, S.A. (1981*b*). Promotion by unsaturated fat of azaserine-induced pancreatic carcinogenesis in the rat. *Cancer Res.*, **41**, 3961–3966.

ROEBUCK, B.D., BAUMGARTNER, K.J., THRON, C.D. & LONGNECKER, D.S. (1984). Inhibition by retinoids of the growth of azaserine-induced foci in the rat pancreas. *J. Natl Cancer Inst.*, **73**, 233–236.

ROEBUCK, B.D., LONGNECKER, D.S., BAUMGARTNER, K.J. & THRON, C.D. (1985). Carcinogen-induced lesions in the rat pancreas: effects of varying levels of essential fatty acid. *Cancer Res.*, **45**, 5252–5256.

ROSENBERG, L., DUGUID, W.P., BROWN, R.A., GREELEY, G., THOMPSON, J.C. & FRIED, G.M. (1983). The effect of cholecystectomy on plasma CCK and pancreatic growth in the hamsters. *Gastroenterology*, **84**, 1289.

ROSENBERG, L., DUGUID, W.P. & BROWN. R.A. (1984). Cholecystectomy stimulates hypertrophy and hyperplasia in the hamster pancreas. *J. Surg. Res.*, **37**, 108–111.

SCARPELLI, D.G., RAO, M.S. & REDDY, J.K. (1984). Studies of pancreatic carcinogenesis in different animal models. *Environ. Health Perspect.*, **56**, 219–227.

SCHAEFFER, B.K., ZURLO, J. & LOCKNECKER, D.S. (1990). Activation of c-Ki-ras not detectable in adenomas or adenocarcinomas arising in rat pancreas. *Mol. Carcinog.*, **3**, 165–170.

SCHLAG, P., ULRICH, H., MERKLE, P., BÖCKLER, R., PETER, M. & HERFARTH, C. (1980). Are nitrite and n-nitroso compounds in gastric juice risk factors for carcinoma in the operated stomach? *Lancet*, **i**, 727–729.

SCHLAG, P., BÖCKLER, R. & PETER, M. (1982). Nitrite and N-nitroso compounds in gastric juice: risk factors for gastric cancer? *Scand. J. Gastroenterol.*, **17**, 145–150.

SOLOMON, T.E. (1986). Trophic effects of pentagastrin on gastrointestinal tract in fed and fasted rats. *Gastroenterology*, **91**, 108–116.

STACE, N.H., PALMER, T.J., VAJA, S. & DOWLING, R.H. (1987). Longterm pancreaticobiliary diversion stimulates hyperplastic and adenomatous nodules in the rat pancreas: a new model for spontaneous tumour formation. *Gut*, **28** (suppl 1), 265–268.

STEWART, I.D., FLAKS, B., WATANAPA, P., DAVIES, P.W. & WILLIAMSON, R.C.N. (1991). Pancreatobiliary diversion enhances experimental pancreatic carcinogenesis. *Br. J. Cancer*, **63**, 63–66.

STOCK-DAMGÉ, C., APRAHAMIAN, M., LHOSTE, E., POUSSE, A., HUMBERT, W., NORIEGA, R. & GRENIER, J.F. (1984). Pancreatic hyperplasia after small bowel resection in the rat: dissociation from endogenous gastrin levels. *Digestion*, **29**, 223–230.

STRUTHERS, B.J., MACDONALD, J.R., DAHLGREN, R.R. & HOPKINS, D.T. (1983). Effects on the monkey, pig and rat pancreas of soy products with varying levels of trypsin inhibitor and comparison with the administration of cholecystokinin. *J. Nutr.*, **113**, 86–97.

TAMURA, Y., HIRADO, M., OKAMURA, Y., MINATO, Y. & FUJII, S. (1977). Synthetic inhibitors of trypsin, plasmin, kallikrein, thrombin $C_1r$, and $C_1$ esterase. *Biochem. Biophys. Acta.*, **484**, 417–422.

TAYLOR, P.R., DOWLING, R.H., PALMER, T.J., HANLEY, D.C., MURPHY, G.M., MASON, R.C. & MCCOLL, I. (1989). Induction of pancreatic tumours by long term duodenogastric reflux. *Gut*, **30**, 1596–1600.

URBAN, E., MICHEL, A.M. & WESER, E. (1982). Dissociation of mucosal mass and adaptive changes in electrolyte, water and sugar transport in rats after intestinal resection. In *Mechanisms of Intestinal Adaptation*, Robinson, J.W.L., Dowling, R.H. & Riecken, E.-O. (eds) pp. 529–547. MTP Press: Lancaster.

VAN KRANEN, H.J., VERMEULEN, E., SCHOREN, L., BAX, J., WOUTERSEN, R.A., VAN LERSEL, P., VAN KREIJL, C.F. & SCHERER, E. (1991). Activation of c-K-ras is frequent in pancreatic carcinomas of Syrian hamsters, but is absent in pancreatic tumours of rats. *Carcinogenesis*, **12**, 1477–1482.

WATANAPA, P., BEARDSHALL, K., CALAM, J. & WILLIAMSON, R.C.N. (1991*a*). Tropic role of enteroglucagon in pancreatic adaptation to subtotal enterectomy. *Br. J. Surg.*, **78**, 917–920.

WATANAPA, P., EFA, E.F., BEARDSHALL, K., CALAM, J., SARRAF, C.E., ALISON, M.R. & WILLIAMSON, R.C.N. (1991*b*). Inhibitory effect of a cholecystokinin antagonist on the proliferative response of the pancreas to pancreatobiliary diversion. *Gut*, **32**, 1049–1054.

WATANAPA, P. & WILLIAMSON, R.C.N. (1992*a*). Surgical palliation for pancreatic cancer: developments during the past two decades. *Br. J. Surg.*, **79**, 8–20.

WATANAPA, P., EGAN, M., DEPREZ, P.H., CALAM, J., SARRAF, C.E., ALISON, M.R. & WILLIAMSON, R.C.N. (1992*b*). The role of cholecystokinin in pancreatic adaptation to massive enterectomy. *Gut*, **33**, 959–964.

WATANAPA, P., FLAKS, B., OZTAS, H., DEPREZ, P.H., CALAM, J. & WILLIAMSON, R.C.N. (1992*c*). Duodenogastric reflux enhances growth and carcinogenesis in rat pancreas. *Br. J. Surg.*, **79**, 791–794.

WATANAPA, P., FLAKS, B., OZTAS, H., DEPREZ, P.H., CALAM, J. & WILLIAMSON, R.C.N. (1992*d*). Inhibitory effect of a cholecystokinin antagonist on pancreatic carcinogenesis after pancreatobiliary diversion. *HPB Surg.*, **5** suppl., 73.

WATANAPA, P., FLAKS, B., OZTAS, H., DEPREZ, P.H., CALAM, J. & WILLIAMSON, R.C.N. (1992*e*). Promoting effect of partial gastrectomy on pancreatic carcinogenesis. *Br. J. Cancer*, **65**, 383–387.

WEBSTER, P.D., SINGH, M., TUCKER, P.C. & BLACK, O. (1972). Effects of fasting and feeding on the pancreas. *Gastroenterology*, **62**, 600–605.

WILLIAMSON, R.C.N. & BAUER, F.L.R. (1978). Evidence for an enterotropic hormone: compensatory hyperplasia in defunctioned bowel. *Br. J. Surg.*, **65**, 736–739.

WILLIAMSON, R.C.N. & RAINEY, J.B. (1984). The relationship between intestinal hyperplasia and carcinogenesis. *Scand. J. Gastroenterol.*, **19** (suppl. 104), 57–76.

WILLIAMSON, R.C.N. (1988). Pancreatic cancer: the greatest oncological challenge. *Br. Med. J.*, **296**, 445–446.

WISNER, J.R.Jr., MCLAUGHLIN, R.E., RICH, K.A., OZAWA, S. & RENNER, I.G. (1988). Effects of L-364,718, a new cholecystokinin receptor antagonist, on camostate-induced growth of the rat pancreas. *Gastroenterology*, **94**, 109–113.

WOUTERSEN, R.A. & VAN GARDEREN-HOETMER, A. (1988). Inhibition of dietary fat promoted development of (pre)neoplastic lesions in exocrine pancreas of rats and hamsters by supplemental selenium and β-carotene. *Cancer Lett.*, **42**, 79–85.

WYNDER, E.L., MABUCHI, K., MARUCHI, N. & FORTNER, J.G. (1973). Epidemiology of cancer of the pancreas. *J. Natl Cancer Inst.*, **50**, 645–667.